# Multivariate analysis and molecular interaction of curcumin with PPARγ in high fructose diet induced insulin resistance in rats

**DOI:** 10.1186/s40064-016-3364-1

**Published:** 2016-10-06

**Authors:** Vasanthi Jayakumar, Shiek S. S. J. Ahmed, Kesavarao Kumar Ebenezar

**Affiliations:** 1 Molecular Physiology Lab, Faculty of Allied Health Sciences, Chettinad Academy of Research and Education, Rajiv Gandhi Salai, Kelambakkam, Tamil Nadu 603103 India; 2 Drug Discovery Lab, Faculty of Allied Health Sciences, Chettinad Academy of Research and Education, Kelambakkam, Tamil Nadu 603103 India

**Keywords:** Insulin resistance, High fructose diet, Curcumin, Antioxidants, Blood glucose, PPARγ, Molecular docking

## Abstract

To investigate the effect of curcumin on the multivariate and docking analysis on peroxisome proliferator activated receptor-γ, the rats were fed with high fructose diet (Group 2) to induce insulin resistance and curcumin was co-administered orally (Group 4) for a period of 8 weeks and measured the biochemical parameters in blood, kidney and liver tissues. The results showed a significant (p ≤ 0.05) increase in the level of creatinine, glucose, insulin, low density lipoprotein, total cholesterol, triglyceride, urea, uric acid, very low density lipoprotein and decreased albumin, high density lipoprotein and total protein level in the blood of Group 2 when compared with Group 1 control rats. Further, analysis on liver and kidney tissues showed a significant decrease in antioxidants, hexokinase and increased glucose 6-phosphatase and fructose 1,6-bisphosphatase, hydroperoxides and TBARS in Group 2 rats. Furthermore, the multivariate and loading coefficient analysis showed that albumin, HDL, catalase, glutathione reductase, hexokinase and vitamin E are the most contributing factors in blood, liver and kidney. Subsequently, molecular docking was carried out to determine the binding efficiency of curcumin as agonist of PPARγ showed high affinity compared to pioglitazone. The histology of liver and kidney were also studied and the administration of curcumin along with fructose protects the organs from the abnormal changes and also prevents the fat accumulation. Overall, these results demonstrate the preventive role of curcumin on diet induced insulin resistant in rats by ameliorating the altered levels of metabolic changes and potential binding of curcumin with PPARγ as agonist in the treatment of insulin resistance.

## Background

Diabetes mellitus is characterized by insulin resistance and an important public health concern or risk with the quality of life worldwide. About 366 million people are affected with diabetes globally and this expected to rise to 552 million by the year 2030 (Bigoniya et al. 2012). India leads the world with 62.4 million people with type 2 diabetes and expected to rise to 101 million by 2030 (Mohan and Anbalagan 2013). In evidence, the prevalence of diabetic has dramatically increasing due to modern lifestyle with increased consumption of high sugar diet especially fructose (Zimmet et al. 2001). Increased fructose mediates oxidative stress (Pasko et al. 2010) which induces insulin resistance (Gaby 2005) leading to the pathogenesis of diabetes and its complications.

Fructose, a highly lipogenic carbohydrate, that produce large amount of hepatic triose-phosphate for fatty acid synthesis and inhibit lipid oxidation which favor fatty acid re-esterification and synthesis of very low density lipoprotein (VLDL) and triglyceride (TG). In addition, metabolic conversion of fructose to triose-phosphate occur independent of insulin in a rapid manner. Thus increased fructose stimulates lipogenesis and TG, which contribute to reduced insulin sensitivity, hepatic insulin resistance, glucose tolerance and oxidative stress (Pasko et al. 2010; Suwannaphet et al. 2010). In diabetes, insulin resistance leads to glucose overload and its underutilization in the liver. Although several therapeutic strategies has been practiced for treatment of diabetes, there are certain limitations due to their high cost and adverse side effects which includes the development of hypoglycemia, weight gain, gastrointestinal disturbances and liver toxicity (Saikia et al. 2011).

Based on the involvement of oxidative stress in complicating diabetes mellitus, efforts are made to determine the suitable anti-diabetic and anti-oxidant therapeutic strategy (Modak et al. 2007; Srinivasan and Ramarao 2007; Spiller and Sawyer 2006) which may suggest to help in the management of diabetes and thus it remain as a large unmet area of possible therapy without less adverse effects. Recently, the natural substances are used as therapeutics in the management of diabetes mellitus. The hypoglycemic effect of herbal extracts has been demonstrated in human and animal models of type 2 diabetes (Patel et al. 2012; Ayyanar et al. 2008). The World Health Organization (WHO) has recommended that traditional medicinal herbs could be further investigated (Modak et al. 2007) and especially the plant medicine that prevent diabetes mellitus because of the likelihood of high compliance and become largely free from side effects (Suwannaphet et al. 2010). One such phytochemical and polyphenol flavonoid of turmeric, curcumin the ginger family (*Zingiberaceae*) has a long history of use in traditional Indian medicine as supplemental herbal diet (Maheshwari et al. 2006). Curcumin is a hydrophobic polyphenol compound extracted from the rhizome of the herb *Curcuma longa* used as food supplement that possess many pharmacological activities including anti-inflammatory (Aggarwal and Harikumar 2009), anti-cancer properties (Ireson et al. 2002), powerful anti-oxidant (Kuhad and Chopra 2007) and as an anti-diabetic agent (Arun and Nalini 2002).

Recent studies have shown that the interaction of the ligand activated transcription factor peroxisome proliferator-activated receptor gamma (PPARγ) with naturally occurring molecules increases insulin sensitivity and have anti-diabetic capacity (Wang et al. 2014). PPARγ is one of three known isoforms, a regulator of lipid and glucose metabolism responsible for metabolic disorders and also act as the molecular target for drugs against several metabolic disorders (Grygiel-Gorniak 2014; Kim and Ahn 2004). Hence, the present study was focused to evaluate the effect of curcumin on biochemical parameters, their potential changes with multivariate analysis and the effect of docking of curcumin with PPARγ on most contributing factors against high fructose diet fed insulin resistance in adult male Wistar rats.

## Methods

### Chemicals and animal model

The chemicals, reagents, fructose and curcumin were purchased from National Scientific Suppliers, Puducherry, India and are of analytical grade. Adult male Wistar rats weighing 120–140 g were purchased from the Sri Venkateshwara Enterprises, Bangalore, India. All the animals were maintained under standard laboratory conditions at temperature 27 ± 2 °C and 12 h light and dark cycles throughout the experimental period. The rats were provided with laboratory chow (VRK Nutritional solution, Chennai) and water ad libitum. All procedures in the study were conducted in accordance with ethics standards of Institutional Animals Ethical Committee (IAEC), Chettinad Academy of Research and Education, Kelambakkam, TN, India.

#### Experimental design

 The rats were divided into four groups with six animals in each.

Group 1: Control rats received normal rat chow diet daily.
Group 2: Rats received 60 % high fructose diet for 8 weeks.
Group 3: Rats received 80 mg curcumin/kg. b. wt. orally for 8 weeks.
Group 4: Rats received 60 % high fructose diet and 80 mg curcumin/kg. b. wt. orally (co-administration) for 8 weeks.

### Induction of insulin resistance in rats

60 % fructose diet was prepared by weighing 60 g of fructose mixed with 40 g of rat chow diet and fed to the rats (Group 2) for a minimum of 8 weeks for the induction of insulin resistance (Xue et al. 2008; Park et al. 2015; Dupas et al. 2016). The fasting blood glucose level was measured in serum (blood was collected from the tail vein using lancet) to assess the induction of insulin resistance every week and the animals with blood glucose concentration more than 200 mg/dl were taken for the study (Wilson and Islam 2012; Tan and Kim 2013).

Curcumin was dissolved in 0.2 % Dimethyl Sulfoxide (DMSO) solution and given orally to rats using an intra-gastric tube (Mathews et al. 2012). Earlier studies have reported that DMSO as a vehicle does not have any toxic effect (Castro et al. 2014; Basnet and Shalko-basnet 2011) even with a high dose of 5 ml/kg b. wt. was tolerable in rats and it does not affect the values when compared with control rats (Gad 2009). At the end of the experimental period, animals were anesthetized and decapitated in order to reduce stress, blood was collected rapidly in tubes containing anticoagulant and without anticoagulant for plasma and serum separation, respectively. The collected blood was centrifuged at 3000 rpm for 20 min. The serum was used for insulin assay and the plasma was used for other biochemical parameters. In addition, liver and kidney were immediately dissected out, washed in ice-cold 0.15 M saline to remove the excess of blood and a part of it was preserved and used for histological studies. The remaining tissues was weighed and homogenized using mortar and pestle to prepare 10 % tissue homogenate using ice-cold tris–Hcl buffer (0.2 M, pH 7.4). The homogenate was centrifuged at 10,000 rpm for 20 min at 4 °C and the supernatant was used for the estimation of antioxidants, lipid peroxidation enzymatic and non-enzymatic assays. All samples were collected early in the morning after animals had fasted overnight (12 h).

#### Analytical procedures

Serum insulin was measured by chemiluminesence immunoassay (CLIA) explained by Marschner et al. (1974). Plasma glucose was estimated using a commercial kit (Sigma Diagnostics (I) Pvt. Ltd., Baroda, India) (Trinder 1969). Also, the plasma urea levels was determined using Fawcett and Scott method (1960), uric acid by Caraway (1955), creatinine by Tietz (1987), albumin and total protein by Reinhold (1953) and lipid profile (Total Cholesterol, TG, HDL, LDL, VLDL) were estimated spectrophotometrically according to the standard procedures using commercially available diagnostic kits (Sigma diagnostic (I) Pvt. Ltd., Baroda. India*).* Further, the levels of antioxidants, lipid peroxides and metabolizing enzymes were studied in the tissue samples of liver and kidney. The antioxidants such as catalase (CAT) (Sinha 1972), glutathione peroxidase (GPx) (Rotruck et al. 1984), super oxide dismutase (SOD) (Kakkar et al. 1984), reduced glutathione (GSH) (Ellman 1959) were determined. Lipid peroxidation was measured as a thiobarbituric acid reacting substances (TBARS) using Niehius and Samuelson (1968), hydroperoxides by Jiang et al. (1992) and total protein was estimated by Lowry et al. (1951). Vitamin E was determined by the method of Baker and Frank et al. (1951) and vitamin C level by the method of Omaye et al. (1979). For metabolizing enzymes, hexokinase activity was determined by the method of Brandstrup et al. (1957), Glucose 6-phosphatase activity by Koida and Oda (1959) and fructose 1,6-bisphosphatase as described by Gancedo and Gancedo (1971).

#### Protein–ligand docking

Molecular docking study was carried out with curcumin as agonist of PPARγ (Lewis et al. 2010). The agonist of PPARγ suggests increasing the concentration of albumin, CAT, GSH and hexokinase (Al-Malki and El Rabey 2015; Panasyuk et al. 2012; Garcia-Fuentes et al. 2010; Dhaunsi et al. 2010), which are the most contributing proteins of PLS-DA analysis. The crystal structure of PPARγ protein was retrieved from Protein Data Bank (3DZY). The solvent molecules and the co-crystallized ligands were removed from the protein structure prior to docking. Docking energy calculations for curcumin with the proteins were done Autodock4 with a grid that accommodates the binding sites cavity for PPARγ in order to allow curcumin to determine its effective confirmation. The protein was used as a rigid model structure with Merck molecular force field (MMFF) used for the scoring function. To test the binding efficiency of curcumin, the co-crystallized ligand pioglitazone (2XKW) was docked into the PPARγ. Different orientations of the ligands were searched and ranked based on their least energy scores.

#### Histology studies: hematoxylin and eosin (H&E) staining

The saline washed liver and kidney tissues were fixed with 10 % formalin solution for the histological examinations. The paraffin embedded tissue sections were stained with H & E were examined and photographed under a light microscope for observation of structural abnormalities (Wagnerberger et al. 2013; Sun et al. 2013).

#### Oil red O staining

For the detection of lipids, portions of liver were rapidly frozen in cryostat and embedded in Tissue-Tek, 3–4 μm cryosections were mounted on the microscope slides and air-dried for 2 h. After fixation in 4 % neutral formaldehyde for 10 min, sections were stained with oil red O (0.5 % oil red O dissolved in propylene glycol) for 10 min at 60 °C. The sliced sections were then counterstained for histopathology (Wagnerberger et al. 2013).

### Statistical analysis

The significance changes in biochemical parameters were analyzed using statistical SPSS (version 21) package. Analysis of variance (ANOVA) was performed to demonstrate a significant difference (p ≤ 0.05) in biochemical parameters between the analyzed groups. Furthermore, the multidimensional data were subjected to partial least square discriminant analysis (PLS-DA) using SIMCA software (Umetrics, Inc., Kinnelon, NJ) to confirm the potential contribution of these biochemical parameters to differentiate the groups from control. Also, the importance of each parameter in the PLS-DA was evaluated by variable importance in the projection (VIP) scores. The score greater than 1 were positively reflects the influence of biochemical parameters on the classification and the correlation analysis was demonstrated to determine the interdependency between the biochemical parameters towards the classification between groups.

## Results and discussion

The present study was designed to explore the effect of oral administration of curcumin against high fructose diet induced insulin resistance in adult male Wistar rats resulted in the following findings: the level of glucose¸ insulin and renal markers were significantly decreased and increased level of antioxidants, HDL with the alterations in the metabolic enzymes in Group 4 compared to Group 2 rats. The average concentration of each parameter was calculated and represented as tables along with their standard deviation (SD). The typical analyses of antioxidants, lipids, lipid peroxidation, metabolizing enzymes and renal markers suggest a critical role of curcumin in preventing insulin resistance in adult male Wistar rats. These findings shows that the co-administration of curcumin possesses a potential antihyperglycemic effect by ameliorating the disturbances caused in the Group 2 insulin resistance induced animals.

### Biochemical variations in blood

Glucose, an abundant molecule and contribute for insulin resistance. Diet high in fructose induce insulin resistance in experimental rats and reduce insulin sensitivity associated with impaired action of hepatic insulin and also glucose disposal from the body (Elliot et al. 2002). At the same time, fructose, a lipogenic sugar, high in diet is independent of insulin action causes increased production of triglycerides leads to lipogenesis rapidly due to unregulated fructose metabolism. This in turn results in insulin resistance which reduces glucose uptake resulting in an increase in the fasting levels of blood glucose and insulin secretion (Basciano et al. 2005; Ramesh and Saralakumari 2012). Statistical analysis of biochemical parameters showed a significant increase in creatinine, glucose, insulin, LDL, total cholesterol, TG, urea, uric acid, VLDL and decreased concentration of albumin, HDL and total protein in Group 2 rats (p ≤ 0.05) when compared with group1 control rats (Table 1). Further, the co-administration of curcumin along with fructose (Group 4) showed the reinstating of most of the biochemical parameters (Table 1).

**Table 1:** Effect of curcumin on glucose, insulin, lipid profile and renal markers in blood of control and experimental rats

Biochemical parameters	Group 1	Group 2	Group 3	Group 4
Fasting blood glucose (mg/dl)	109.53 ± 9.08	207.63 ± 9.90*	108.64 ± 7.86	114.11 ± 12.74^#^
Fasting insulin (µU/ml)	0.17 ± 0.02	0.99 ± 0.14*	0.24 ± 0.02	0.33 ± 0.02^#^
Total Cholesterol (mg/dl)	142.16 ± 41.23	263.33 ± 36.14*	175.83 ± 28	162.16 ± 26.23^#^
Triglycerides (mg/dl)	106.5 ± 8.19	269.16 ± 28.18*	99.66 ± 13.1	102.66 ± 18.99^#^
HDL (mg/dl)	47.16 ± 11.73	37.5 ± 13.69*	82.66 ± 16.08	95.16 ± 18.87^#^
LDL (mg/dl)	73.7 ± 33.08	172 ± 43.3*	73.23 ± 40.39	46.46 ± 34.45^#^
VLDL (mg/dl)	21.5 ± 1.54	53.83 ± 5.63*	19.93 ± 2.62	20.53 ± 3.79^#^
Total protein (g/dl)	11.83 ± 0.55	9.14 ± 0.90*	11.08 ± 0.78	10.90 ± 0.47^#^
Albumin (g/dl)	3.33 ± 0.17	2.19 ± 0.19*	3.30 ± 0.28	3.41 ± 0.32#
Urea (mg/dl)	10.79 ± 1.49	16.11 ± 3.60*	10.83 ± 1.22	10.37 ± 0.93^#^
Uric acid (mg/dl)	12.05 ± 3.75	22.90 ± 6.10*	12.99 ± 3.93	13.03 ± 0.62^#^
Creatinine (mg/dl)	0.81 ± 0.01	1.12 ± 0.13*	0.87 ± 0.02	0.82 ± 0.07^#^

Values are represented as mean ± SD, n = 6, p < 0.05, comparisons are made between * Group 1 versus Group 2; ^#^ Group 2 versus Group 4

Our results showed that curcumin is a potent hypolipidemic and renoprotective agent that reduces the lipogenesis and alter the lipogenic enzymes which regulate the homeostatic level of lipids, increase the uptake of glucose peripherally and prevent the changes in lipid metabolism that caused by the administration of high fructose diet.

### Biochemical variations in tissue

In liver, hexokinase is an important regulator of glucose storage and disposal whereas in pancreas it regulate glycolytic rate and play a central role in control of glucose stimulated insulin secretion (O’Doherty et al. 1999). Insulin resistance and lipid peroxidation has been developed with abnormal increase in the production of free radicals and simultaneous reduction of the antioxidants leads to the damage of cellular organelles and enzymes (Maritim et al. 2003). The analysis with kidney and liver tissues showed significant (p ≤ 0.05) decrease in CAT, GPx, hexokinase, hydroperoxides, GSH, SOD, vitamin C and E and increased glucose 6-phosphatase, fructose 1,6-bisphosphatase and TBARS in Group 2, when compared with Group 1 rats (Tables 2, 3). In Group 4 rats, the administration of curcumin along with fructose showed an effective contribution of curcumin in changing the antioxidants and metabolic enzymes (Tables 2, 3). The salubrious effect may be due to co-administration of curcumin along with high fructose diet prevented the increase in the level of antioxidants and decrease in the level of TBARS and lipid hydroperoxides with altered carbohydrate metabolizing enzymes towards the glucose metabolism which help in regulating the homeostasis.

**Table 2:** Influence of curcumin on metabolizing enzymes, antioxidants and lipid peroxides in kidney of control and experimental rats

Biochemical parameters	Group 1	Group 2	Group 3	Group 4
Hexokinase (mM of glucose phosphorylated/h/mg protein)	4.67 ± 0.16	3.34 ± 0.18*	4.76 ± 0.13	5.62 ± 0.26^#^
Glucose 6-phosphatase (mM of inorganic phosphorous liberated/min/mg protein)	28.38 ± 1.72	46.27 ± 2.06*	26.58 ± 1.75	37.19 ± 2.53^#^
Fructose 1,6-bis phosphatase (mM of inorganic phosphorous liberated/h/mg protein)	11.26 ± 1.88	26.97 ± 2.21*	20.69 ± 1.37	18.91 ± 5.58^#^
Catalase (units/mg of protein)	58.55 ± 0.76	17.53 ± 1.64*	49.44 ± 0.91	70.42 ± 0.69^#^
Super oxide dismutase (units/mg protein)	1518.34 ± 8.72	678.73 ± 10.71*	1345.78 ± 16.94	1134.16 ± 11.86^#^
Glutathione peroxidase (units/mg protein)	169.20 ± 1.0	17.53 ± 1.0*	102.94 ± 1.95	124.07 ± 2.18^#^
Vitamin C (µM/mg of tissue)	1.80 ± 0.04	0.72 ± 0.06*	1.8 ± 0.04	1.27 ± 0.03^#^
Vitamin E (µM/mg of tissue)	4.03 ± 0.12	1.35 ± 0.04*	3.52 ± 0.05	2.63 ± 0.07^#^
Glutathione reductase (mg/100 g of tissue)	1.40 ± 0.01	0.40 ± 0.04*	1.36 ± 0.05	0.80 ± 0.01^#^
Thiobarbituric acid reactive substances (mM/100 g tissue)	0.14 ± 0.02	0.34 ± 0.02*	0.12 ± 0.01	0.08 ± 0.003^#^
Hydroperoxides (mM/100 g of tissue)	540.10 ± 10	1271.17 ± 354.15*	517.81 ± 46.55	640.24 ± 73.76^#^

Values are represented as mean ± SD, n = 6, p < 0.05, Comparisons are made between * Group 1 versus group 2; ^#^ Group 2 versus Group 4

**Table 3:** Influence of curcumin on metabolizing enzymes, antioxidants and lipid peroxides in liver of control and experimental rats

Biochemical parameters	Group 1	Group 2	Group 3	Group 4
Hexokinase (mM of glucose phosphorylated/h/mg protein)	4.62 ± 0.16	2.59 ± 0.27*	3.62 ± 0.23	5.38 ± 0.30^#^
Glucose 6-Phosphatase (mM of inorganic phosphorous liberated/min/mg protein)	0.24 ± 0.03	5.69 ± 0.30*	0.24 ± 0.03	1.59 ± 0.15^#^
Fructose 1,6-bis phosphatase (mM of inorganic phosphorous liberated/h/mg protein)	116.25 ± 4.54	167.05 ± 11.10*	131.50 ± 35.10	140.39 ± 5.26^#^
Catalase (Units/mg of protein)	108.67 ± 1.81	69.10 ± 2.06*	108.27 ± 1.85	107.82 ± 1.66^#^
Super oxide dismutase (Units/mg protein)	1526.39 ± 18.29	685.25 ± 4.44*	1337.80 ± 11.87	1135.74 ± 8.20^#^
Glutathione peroxidase (Units/mg protein)	142.00 ± 1.86	21.48 ± 1.11*	93.34 ± 1.81	137.81 ± 2.32^#^
Vitamin C (µM/mg of tissue)	1.60 ± 0.06	0.70 ± 0.02*	1.6 ± 0.06	1.15 ± 0.02^#^
Vitamin E (µM/mg of tissue)	1.56 ± 0.03	0.83 ± 0.04*	1.54 ± 0.04	2.22 ± 0.008^#^
Glutathione reductase (mg/100 g of tissue)	1.59 ± 0.04	0.06 ± 0.003*	1.43 ± 0.04	1.06 ± 0.04^#^
Thiobarbituric acid reactive substances (mM/100 g tissue)	0.05 ± 0.004	0.31 ± 0.03*	0.04 ± 0.006	0.20 ± 0.004^#^
Hydroperoxides (mM/100 g of tissue)	737.12 ± 63.57	1115.18 ± 223.04*	599.56 ± 71.90	779.10 ± 100.92^#^

Values are represented as mean ± SD, n = 6, p < 0.05, comparisons are made between * Group 1 versus Group 2; ^#^ Group 2 versus Group 4

### Multivariate analysis

**Fig. 1:**
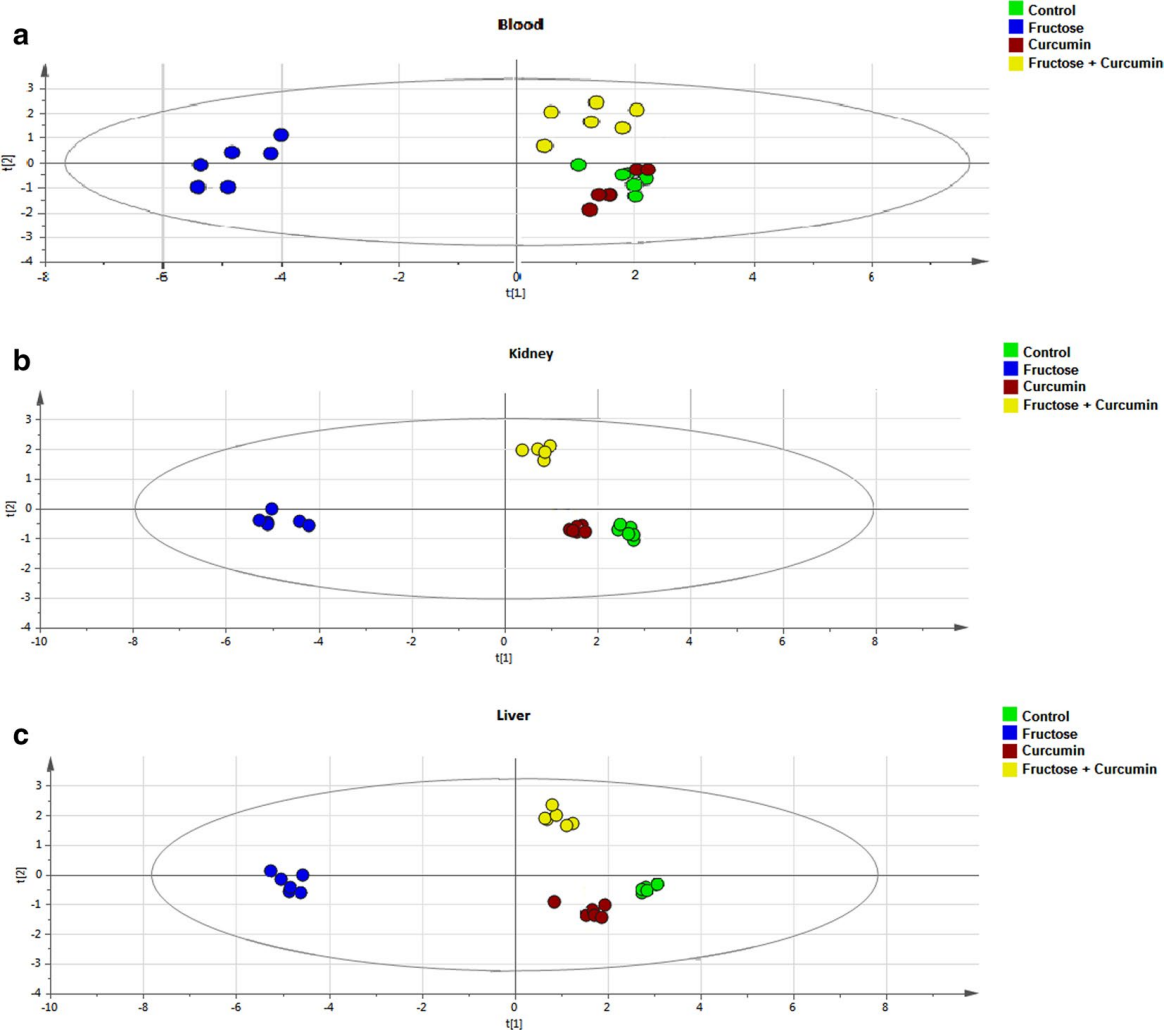
Multivariate PLS-DA analysis. Blood (**a**), kidney (**b**) and liver (**c**) shows a significant differentiation (p ≤ 0.05) between the groups. The observations were coded according to groups: *green* control; *blue* fructose; *red* curcumin; *yellow* fructose + curcumin

**Fig. 2:**
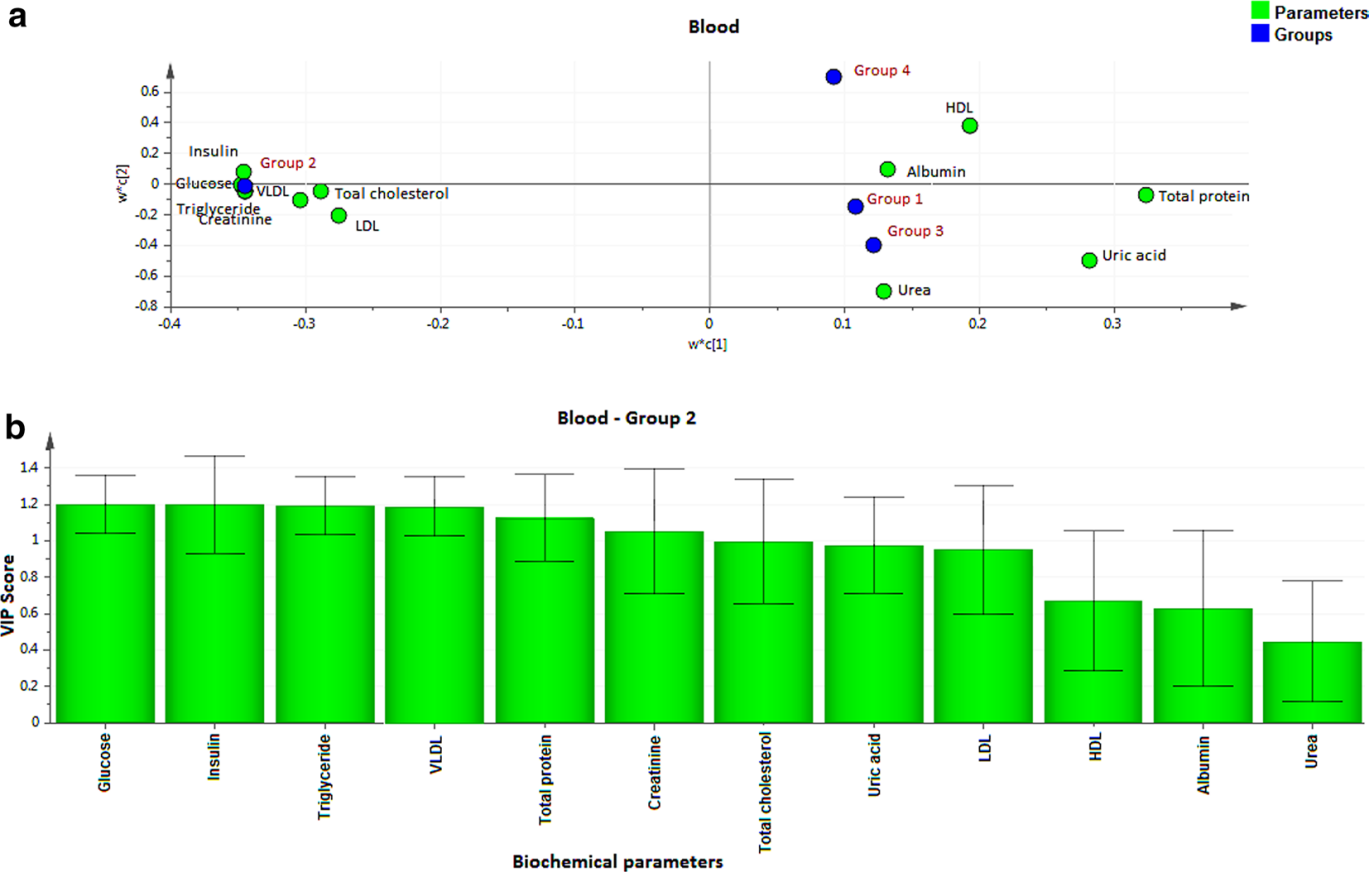
Loading coefficient and VIP plot for blood parameters of Group 2 rats. The loading coefficient map showing (**a**—blood) that insulin, glucose, VLDL, total cholesterol, LDL, triglyceride and creatinine were predominantly responsible for the classification of groups. **b** The VIP scores for the biochemical parameters analyzed in (Group 2 blood) showing glucose, insulin, triglyceride, creatinine and VLDL with VIP ≥ 1

**Fig. 3:**
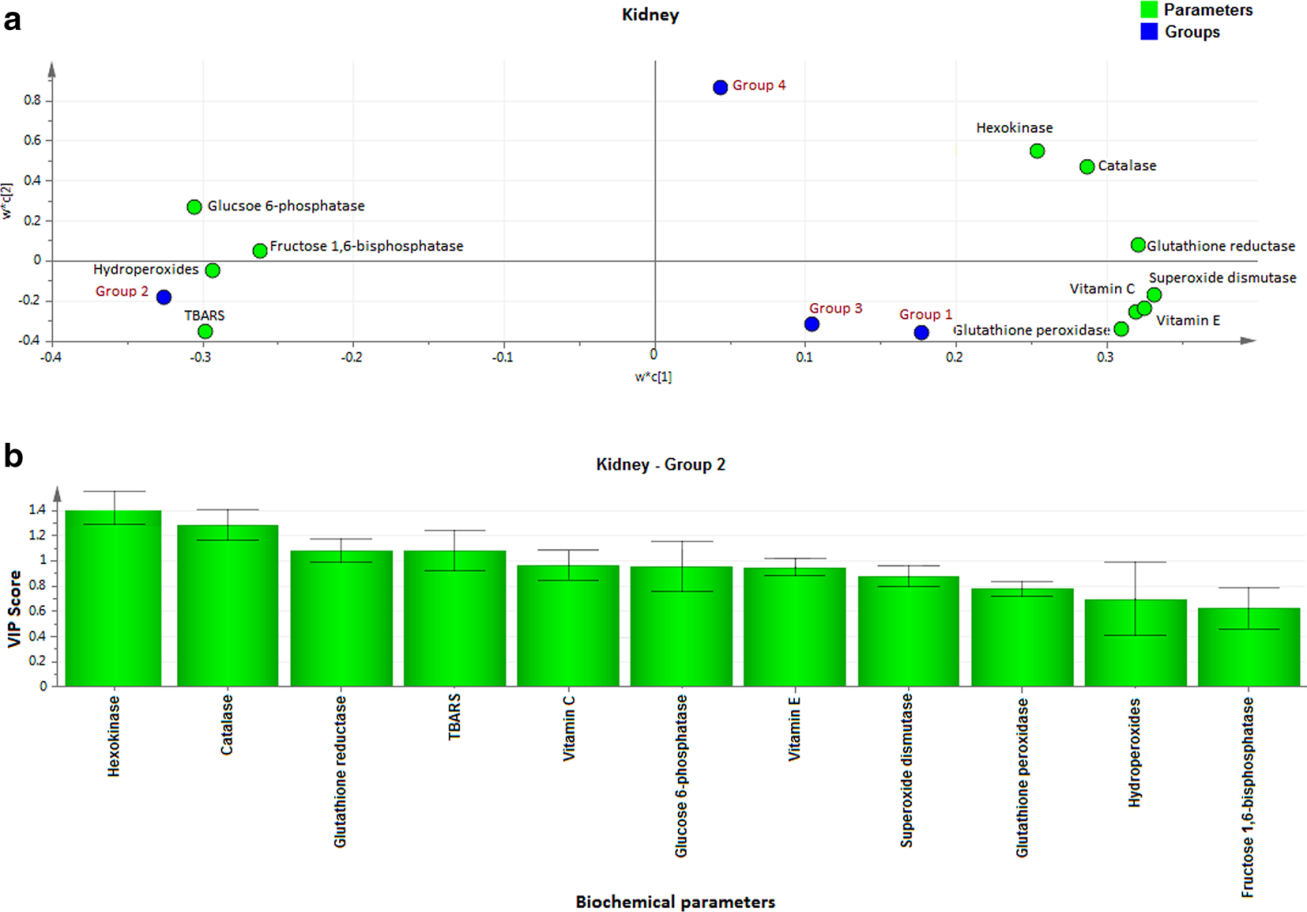
Loading coefficient and VIP plot for kidney parameters of Group 2 rats. The loading coefficient map showing (**a**—kidney) that glucose 6-phosphatase, fructose 1,6-bisphosphatase, hydroperoxides and TBARS were predominantly responsible for the classification of groups. **b** The VIP scores for the biochemical parameters analyzed in (Group 2) kidney showing hexokinase, catalase, glutathione reductase and TBARS with VIP ≥ 1

**Fig. 4:**
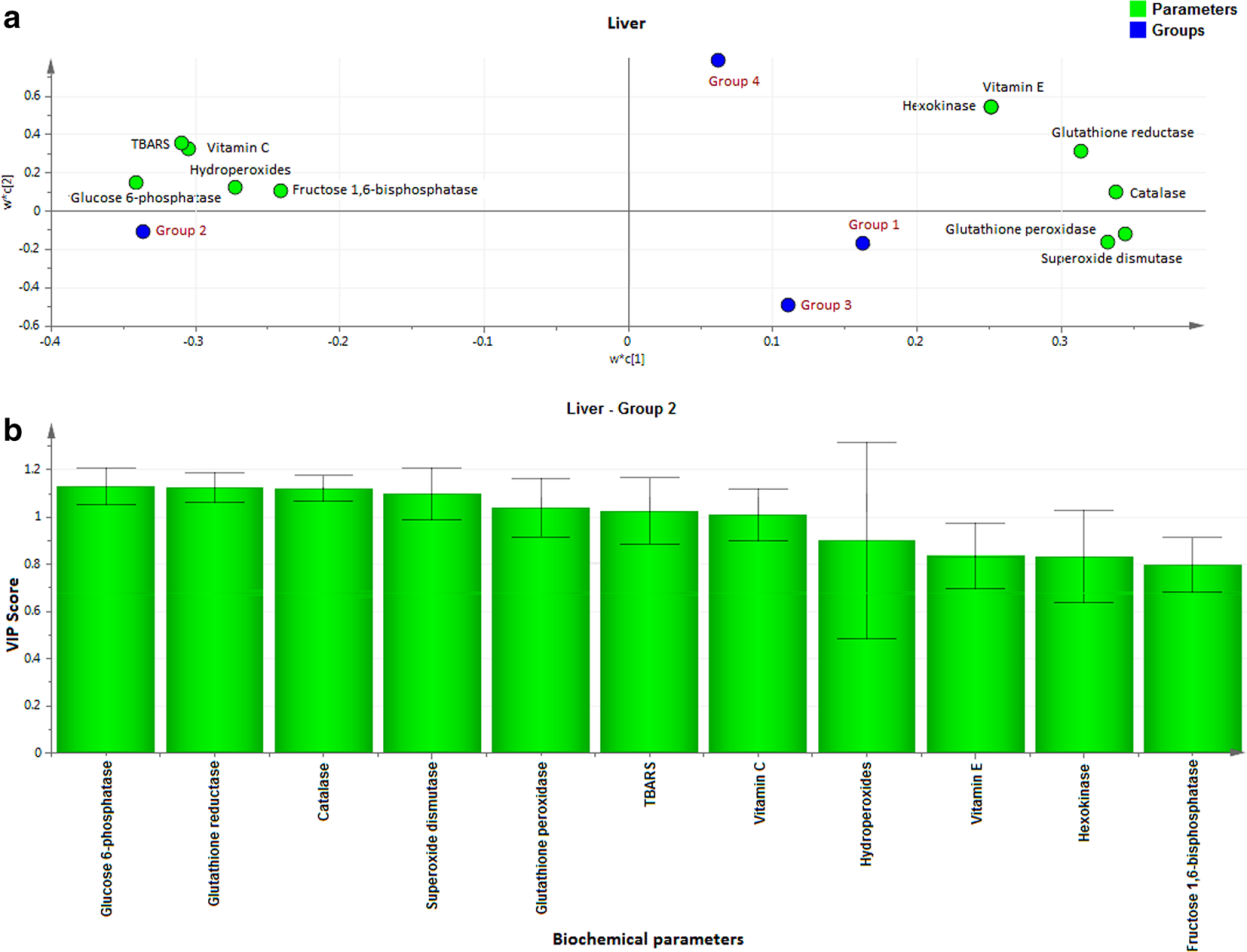
Loading coefficient and VIP plot for liver parameters of Group 2 rats. The loading coefficient map showing (**a**—liver) that glucose 6-phosphatase, fructose 1,6-bisphosphatase, hydroperoxides, vitamin C and TBARS were predominantly responsible for the classification of groups. **b** The VIP scores for the biochemical parameters analyzed in (Group 2) liver showing glucose 6-phosphatase, glutathione reductase, catalase, superoxide dismutase, glutathione peroxidase and TBARS with VIP ≥ 1

**Fig. 5:**
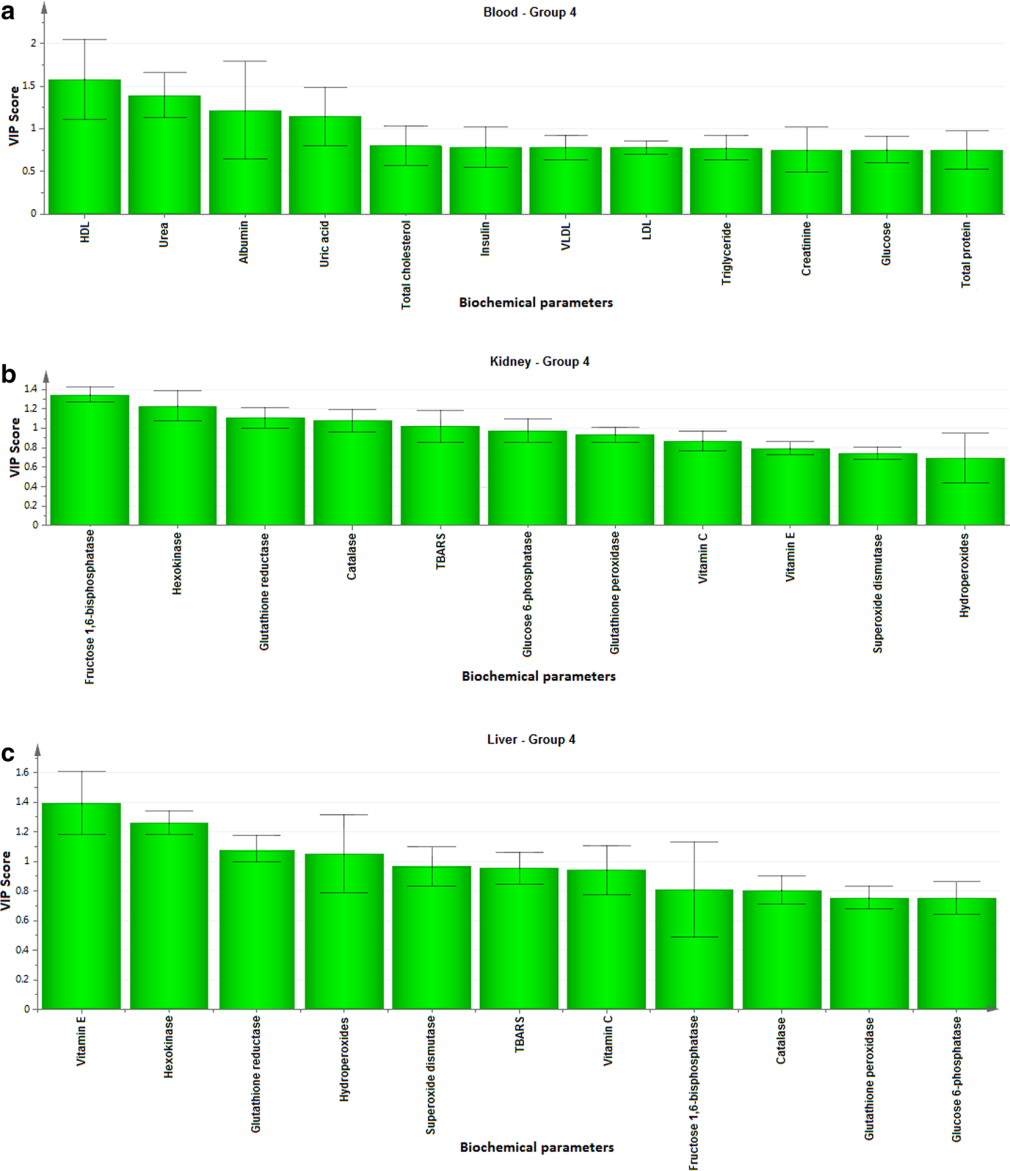
VIP score plots for blood, kidney and liver parameters of Group 4 rats. **a** The VIP scores for the biochemical parameters of blood analyzed in Group 4 showing that HDL, urea, albumin and uric acid with VIP ≥ 1. **b** The VIP scores for the Group 4 kidney showing fructose 1,6-bisphosphatase, hexokinase, glutathione reductase and catalase with VIP ≥ 1. **c** The VIP scores Group 4 liver showing that vitamin E, hexokinase, glutathione reductase and hydroperoxides with VIP ≥ 1

To explore the biochemical multidimensional data, unsupervised statistical method was executed between the groups. The PLS-DA plots’ for blood, kidney and liver showed a clear differentiation of Group 2 from others (Fig. 1a–c). The loading coefficient map of blood (Fig. 2a) indicate a significantly elevated concentrations of creatinine, glucose, insulin, LDL, total cholesterol, TG and VLDL which shows that these factors are predominantly responsible for the separation of Group 2 from other groups. Of these factors, glucose, insulin, creatinine, total cholesterol, TG and VLDL scored ≥1 in VIP plot (Fig. 2b), indicate the potential contribution for insulin resistance. Further, the loading coefficient of kidney (Fig. 3a), biochemical parameters showed that TBARS is the most contributing factors for insulin resistance with the VIP score ≥1 (Fig. 3b). In liver (Fig. 4a), the glucose 6-phosphatase, TBARS and vitamin C were identified as the most contributing factors for insulin resistance with VIP score ≥1 (Fig. 4b). Similarly, the separation of Group 4 samples was attributed to albumin and HDL as the major protecting factor of insulin resistance determined by VIP plot (Fig. 5a). Subsequent analysis showed CAT, GSH, hexokinase are the major protective components of kidney (Fig. 5b) and GSH, hexokinase and vitamin E are the important protective factor of liver from insulin resistance (Fig. 5c). Overall, these biochemical changes confirm the likely importance of fructose in causing insulin resistance.

### Correlation analyses

**Fig. 6:**
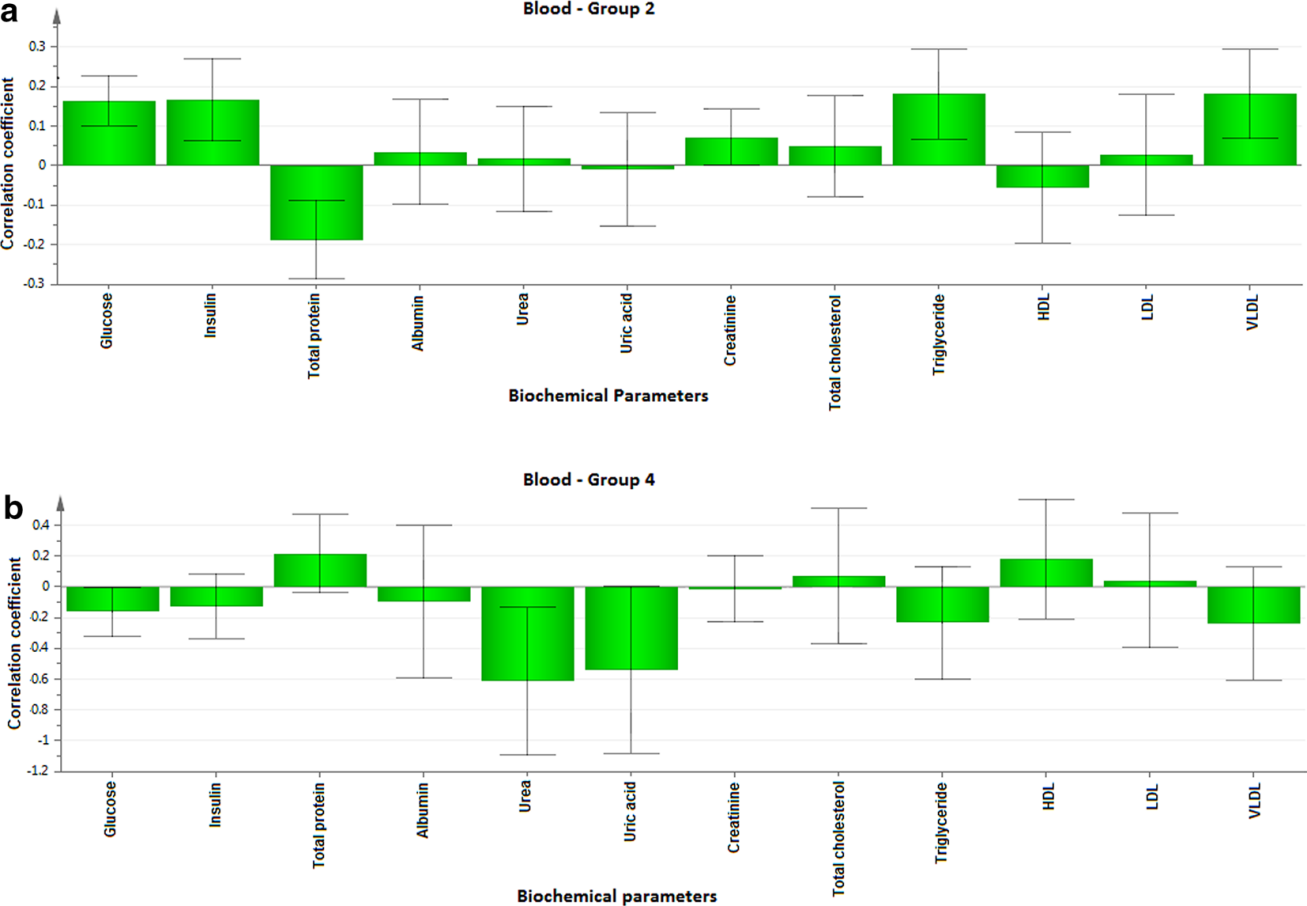
Correlation co-efficient plot for blood parameters of Group 2 rats versus Group 4 rats. Correlation coefficient plots for the blood parameter in the Group 2 (**a**) and Group 4 rats (**b**) shows the association between biochemical parameter with the Group 2 and 4 rats, respectively. The biochemical parameter with *positive value* represents positively correlated and the *negative value* represents the negative association to the analyzed rat

**Fig. 7:**
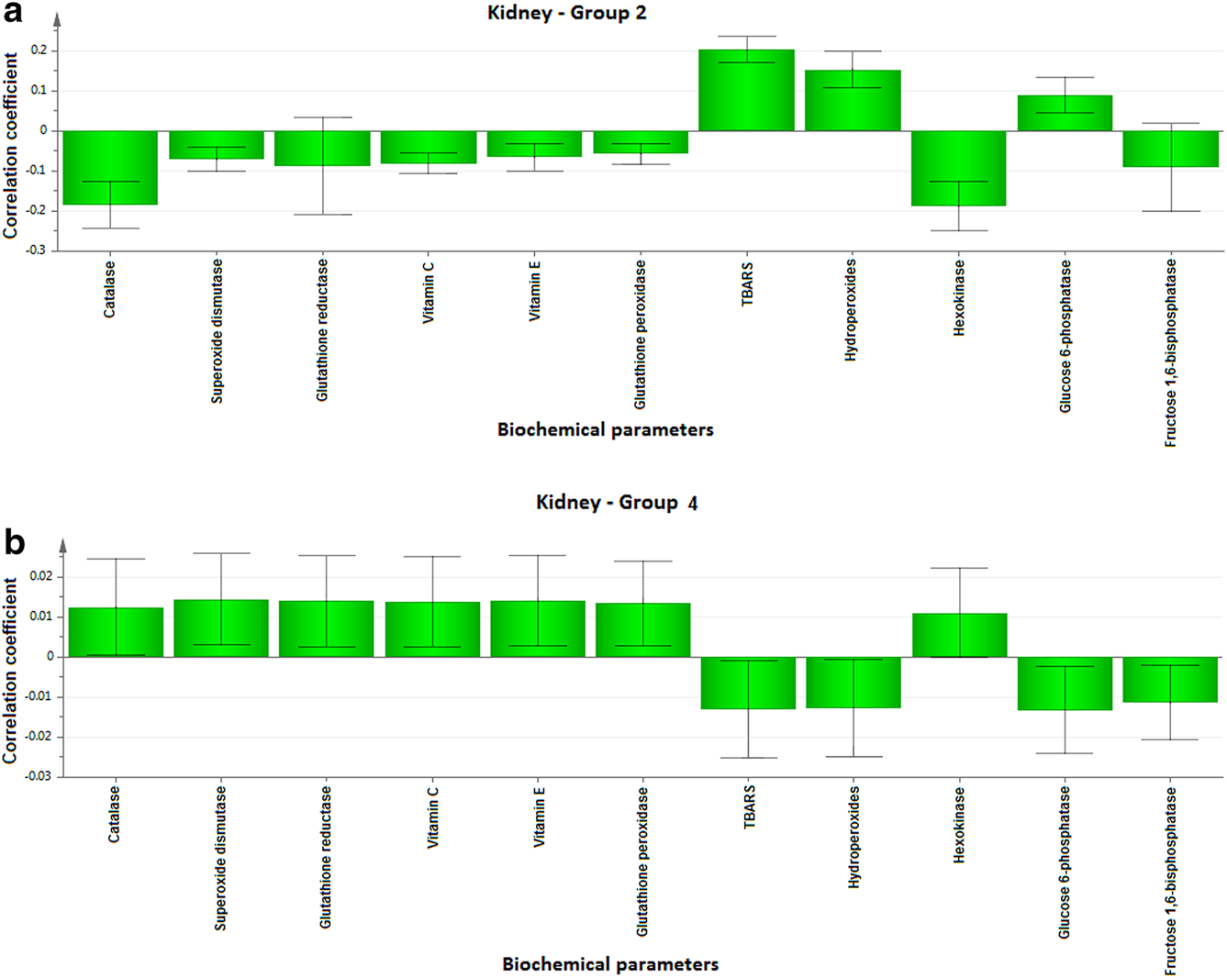
Correlation co-efficient plot for kidney parameters of Group 2 versus Group 4 rats. Correlation coefficient plots for the kidney parameters in the Group 2 (**a**) and Group 4 rats (**b**) shows the association between biochemical parameter with the Group 2 and Group 4 rats, respectively. The biochemical parameter with *positive value* represents positively correlated and the *negative value* represents the negative association to the analyzed rat

**Fig. 8:**
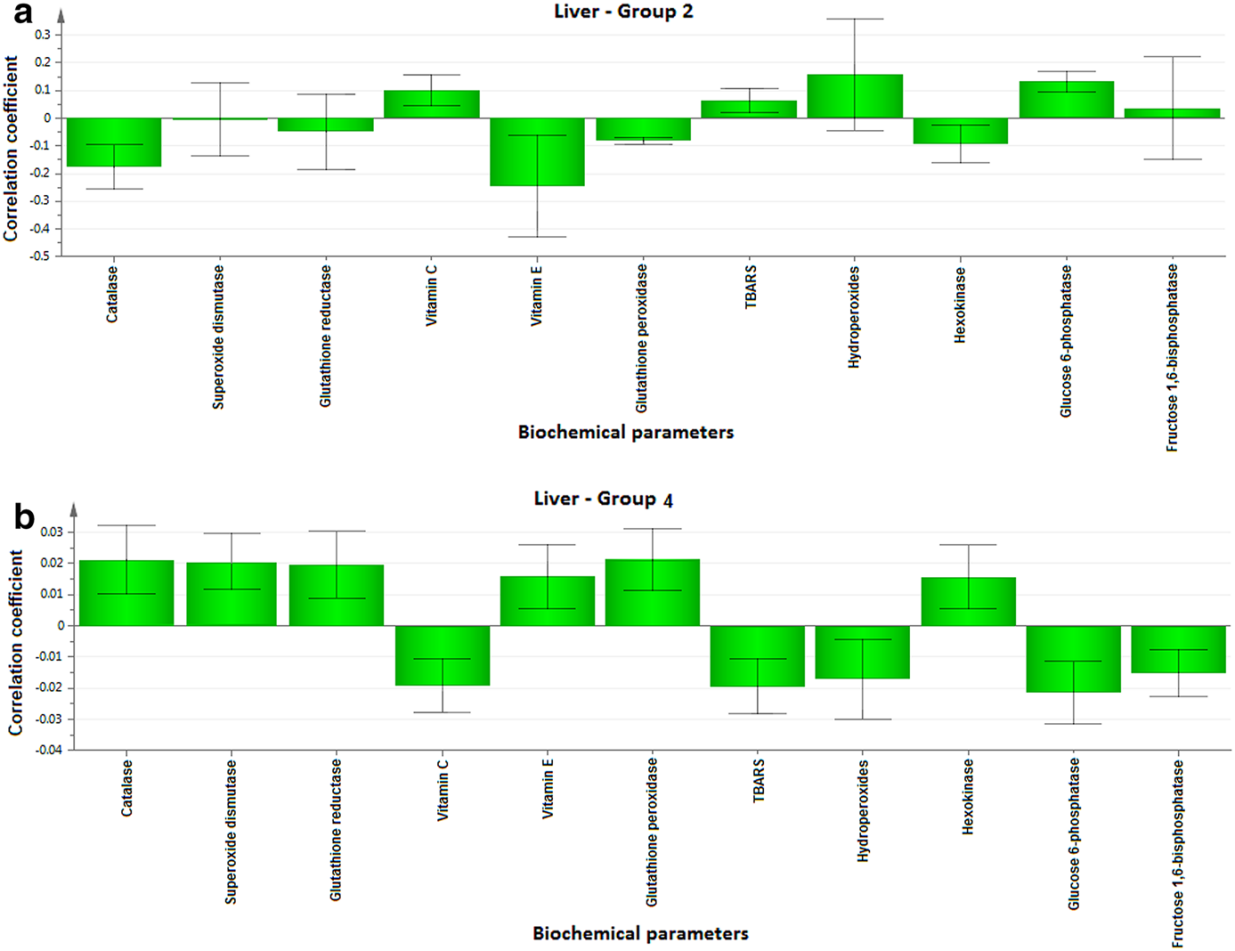
Correlation co-efficient plot for liver parameters of Group 2 versus Group 4 rats. Correlation coefficient plots for the liver parameters in the Group 2 (**a**) and Group 4 rats (**b**) shows the association between biochemical parameter with the Group 2 and Group 4 rats, respectively. The biochemical parameter with *positive value* represents positively correlated and the *negative value* represents the negative association to the analyzed rat

Pearson correlation analysis was carried for Group 2 and 4 rats on the major contributing biochemical factors (VIP score ≥1) in blood and tissues. In Group 2 blood (Fig. 6a), albumin, creatinine, glucose, insulin, total cholesterol, TG, and VLDL were positively associated with insulin resistance. However, reinstation of these molecules were noticed (Fig. 6b) except LDL and total cholesterol in response to co-administration of curcumin (Group 4). Similarly, TBARS of kidney (Fig. 7) and glucose 6-phosphatase, TBARS and vitamin C of liver (Fig. 8) was reinstated in Group 4 compared to Group 2 rats. In addition to these changes, most of the molecules such as albumin and HDL-Cholesterol of blood, CAT, GSH, hexokinase of kidney, GSH, hexokinase and vitamin E of liver showed a positive association in Group 4 rats upon co-administration with curcumin.

### Molecular docking

**Fig. 9:**
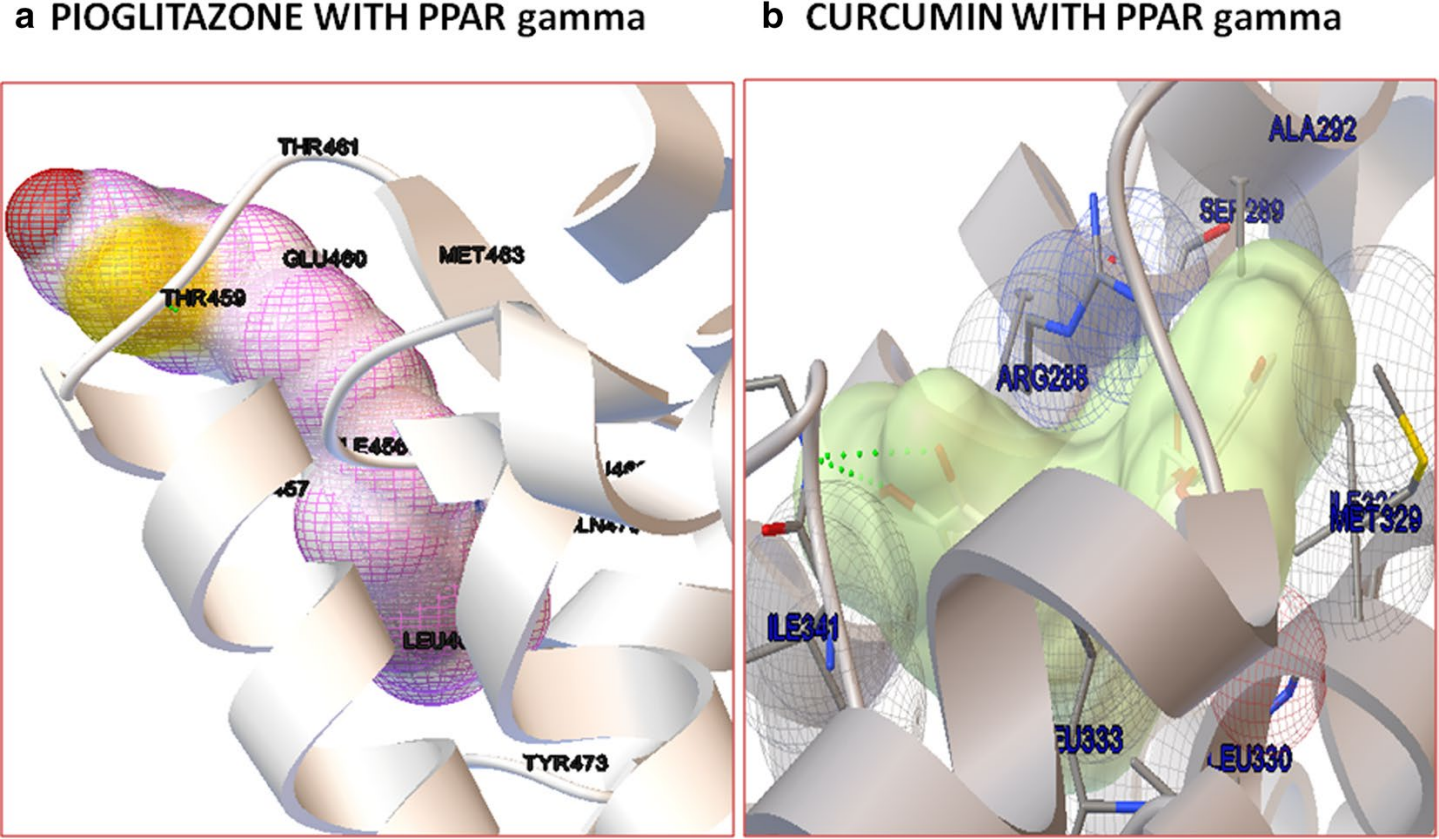
Molecular docking study of PPAR gamma. **a** The binding efficiency of pioglitazone with PPAR gamma. **b** The binding efficiency of curcumin with PPAR gamma of particular aminoacids in the binding sites

PPARγ, a ligand activated transcription factor that regulate various metabolic processes especially lipid and glucose homeostasis (Grygiel-Gorniak 2014; Khamkar et al. 2013; Setzer and Ogungbe 2012). As a molecular target for drugs against several metabolic disorders, PPARγ improves glucose homeostasis by regulating the expression hexokinase and inhibiting G6Pase and also regulates the action of insulin. Hence the molecular docking study was carried out to investigate the binding efficiency of curcumin as an agonist for PPARγ using Autodock4 (Prashantha Kumar et al. 2012). A molecular docking study was carried out to investigate the binding efficiency of curcumin as an agonist for PPARγ and to examine the most contributing factors on the experimental groups. The docking accuracy was evaluated in terms of the root mean square deviation (RMSD) and the prediction was considered successful if the RMSD value was less than 1.8 Å. The best ten energy poses for the curcumin against protein target was determined. The results are ranked according to least binding energies for score. The top ranked binding efficiency of curcumin with PPARγ showed −9.44 kcal/mol (Table 4). Curcumin showed better interaction with PPARγ at their active site. For Instance, Ile(341), Arg(288), Ser(289), Ala(292), Leu(333), Ile(326), Leu(330) and Met(329) contributes curcumin to binding region. Similarly, pioglitazone showed least binding energy of −7.92 which is comparatively high than curcumin. The agonist of PPARγ suggested an increase in the concentration of albumin, hexokinase, CAT and GSH which are the most contributing proteins of PLS-DA analysis (Fig. 9). Overall, the docking results showed that curcumin is potentially involved in binding with the PPARγ as agonist that increase the concentration of most contributing factors that are associated with diabetes.

**Table 4:** Molecular docking of curcumin with PPAR gamma and pioglitazone

Rank	Binding energy of curcumin with PPARγ	Binding energy of curcumin with pioglitazone
1.	−9.44	−7.92
2.	−9.53	−7.42
3.	−9.89	−7.3
4.	−7.55	−8.42
5.	−10.04	−9.35
6.	−5.6	−6.11
7.	−9.98	−8.27
8.	−8.94	−8.64
9.	−10.16	−6.7
10.	−8.94	−9.49

### Histology

**Fig. 10:**
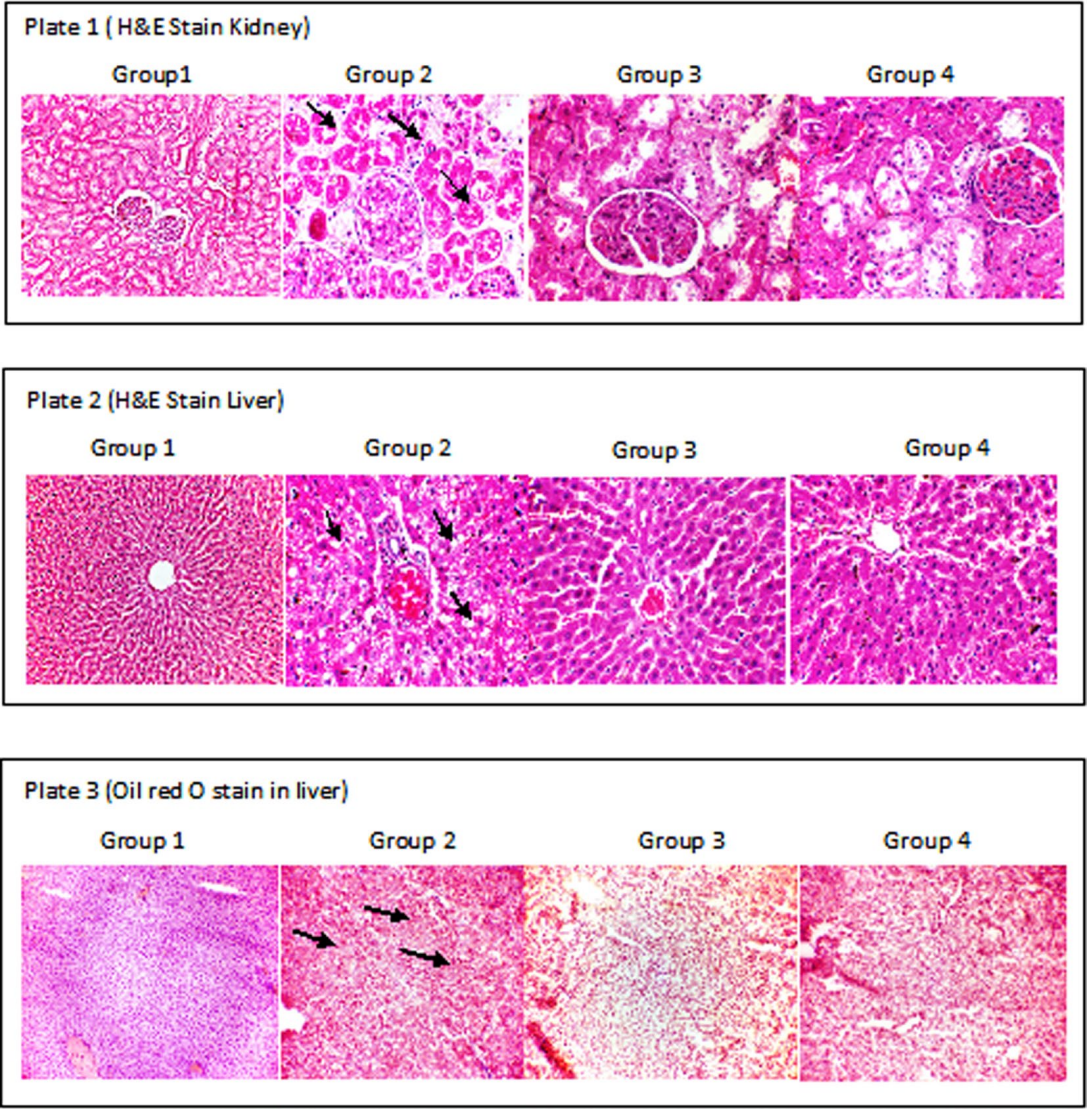
Effect of curcumin on histological changes in liver and kidney of control and experimental rats. *Plate 1* shows the representative photographs of H&E stain on kidney (×400); *Plate 2* shows the representative photographs of the H&E stain on liver (×100); *Plate 3* shows the representative photographs of the Oil Red O stain on liver tissue (×100). *Group 1* control; *Group 2* fructose; *Group 3* curcumin; *Group 4* curcumin + fructose

The histopathology of liver and kidney of control and experimental rats were studied (Fig. 10). Several studies showed that high fat and high fructose diets induce insulin resistance and tissue damage. Excess fructose diet increases the lipid synthesis and metabolic diseases which cause lipotoxic cellular dysfunction and cause accumulation of lipids induces damages in the liver and kidney (Abo-youssef 2015; Hao et al. 2015; De Castro et al. 2013). The histology of kidney stained with H & E. Group 1 shows the normal architecture of the kidney (Plate 1). In Group 2 rats administered high fructose diet, necrosis of the proximal convoluted tubules (tubular necrosis) was observed. No characteristic histological changes were seen in Group 3 administered curcumin alone. A mild glomerular congestion with less cloudy changes of proximal tubule was seen in Group 4 rats co-administered with curcumin when compared to Group 2 rats. Also, the histological findings of liver in Group1 control group depicts the normal architecture of liver and Group 2 rats fed with high fructose diet shows portal congestion with periportal steatosis (fatty change) confirms the pathological condition. Group 3 curcumin administered rats showed no histological changes and Group 4 rats co-administered with high fructose diet and curcumin resulted in reduced centrilobular congestion with mild micro vesicular steatosis when compared to Group 2 rats. In addition, the Oil red O Staining showed in Plate 3 represents that there were no lipid droplets in the Group 1 and Group 3 rats whereas Group 2 rats showed significant infiltration of lipid accumulated in portal centrilobular cells. The Group 4 rats express the recovery of fatty changes in the liver with the co-administration of curcumin. Previous studies reported the accumulation of lipids, hepato-cellular damage in liver and glomerular congestion of kidney in high fat and high fructose diet fed rats (Lozano et al. 2016; Lee et al. 2015). These reports correlates with our study in Group 2, high fructose diet fed rats with those observations. The histological analysis of liver and kidney revealed that the co-administration of curcumin protects the organs from the abnormal changes caused by the high fructose diet and thus co-administration of curcumin along with fructose effectively prevent the damages caused with high fructose diet induced insulin resistance in rats.

## Conclusion

In conclusion, the present study demonstrate that curcumin posses potential anti-hyperglycemic effect through increased insulin production associated with subsequent increase in the activity of antioxidants and glycolytic enzyme, decrease in the activity of gluconeogenic enzymes, alterations in lipids and renal markers. This study revealed that co-administration of curcumin along with fructose protects the metabolic abnormalities caused by high fructose diet and oxidative stress in insulin resistance induced rats. Also, the multivariant analysis highlighted the most contributing factors like albumin, hexokinase, CAT and GSH which has been enhanced by docking with curcumin to PPARγ as agonist. Overall, the current study suggests that curcumin act as a potent regulator of PPARγ thereby alters the most contributing factors that protect against metabolic disorders leading to diabetes mellitus. Further studies are underway to establish the role of curcumin as agonist for PPARγ with gene expression in controlling diabetic complications.
